# Comparative characterization of antibody responses induced by Ad5-vectored spike proteins of emerging SARS-CoV-2 VOCs

**DOI:** 10.1038/s41392-022-01065-0

**Published:** 2022-07-29

**Authors:** Busen Wang, Jinghan Xu, Shipo Wu, Zhe Zhang, Zhenghao Zhao, Jun Zhang, Ling Fu, Xiaodong Zai, Yudong Wang, Guanying Zhang, ZhengShan Chen, Yi Chen, Hancong Sun, Xiaohong Song, Jinlong Zhang, Lianhui Zhu, Lihua Hou, Wei Chen

**Affiliations:** Beijing Institute of Biotechnology, No. 20 Dongdajie Street, Fengtai District, 100071 Beijing, China

**Keywords:** Infectious diseases, Vaccines

## Abstract

Highly divergent SARS-CoV-2 variants have continuously emerged and spread around the world, and updated vaccines and innovative vaccination strategies are urgently needed to address the global SARS-COV2 pandemic. Here, we established a series of Ad5-vectored SARS-CoV-2 variant vaccines encoding multiple spike proteins derived from the Alpha, Beta, Gamma, Epsilon, Kappa, Delta and Omicron lineages and analyzed the antibody immune responses induced by single-dose and prime-boost vaccination strategies against emerging SARS-CoV-2 variants of concern (VOCs). Single-dose vaccination with SARS-CoV-2 variant vaccines tended to elicit the optimal self-matched neutralizing effects, and Ad5-B.1.351 produced more broad-spectrum cross-neutralizing antibodies against diverse variants. In contrast, prime-boost vaccination further strengthened and broadened the neutralizing antibody responses against highly divergent SARS-CoV-2 variants. The heterologous administration of Ad5-B.1.617.2 and Ad5-B.1.429 to Ad5-WT-primed mice resulted in superior antibody responses against most VOCs. In particular, the Omicron spike could only stimulate self-matched neutralizing antibodies with infrequent cross-reactivities to other variants used in single-dose vaccination strategies; moreover, with prime-boost regimens, this vaccine elicited an optimal specific neutralizing antibody response to Omicron, and prompted cross-antibody responses against other VOCs that were very similar to those obtained with Ad5-WT booster. Overall, this study delineated the unique characteristics of antibody responses to the SARS-CoV-2 VOC spikes with the single-dose or prime-boost vaccination strategies and provided insight into the vaccine development of next SARS-CoV-2 VOCs.

## Introduction

The rapid growth and spread of various SARS-CoV-2 variants have aroused great attention due to their increased viral transmission and stronger resistance to neutralizing antibodies compared with the original SARS-CoV-2.^1^ As of January 25, 2022, the B.1.1.7, B.1.351, P.1, B.1.617.2, and B.1.1.529 variants were classified as variants of concern (VOCs) by the World Health Organization or Centers for Disease Control and Prevention in the United States. In addition, the B.1.429 and B.1.617.1 variants were prevalent in California and India, respectively, and were once included as VOCs. Each variant has many mutations in the crucial spike protein, which could lead to antigenic changes detrimental to vaccine protection. Most of the variants have a D614G mutation in the spike (S) protein, which alters the S protein to an ACE-2-binding and fusion-competent conformation and thereby increases viral transmission.^2^ Among the above-mentioned variants, B.1.1.7 has 10–13 mutations, including the N501Y mutation in the receptor-binding domain (RBD) and deletions at aa 69-70 and 144/145 in the N-terminal domain (NTD);^3^ the B.1.351 variant has ten mutations, including the K417N, E484K, and N501Y mutations in the RBD and the deletion of aa 244-246 in the NTD;^3^ the P.1 variant features ten substitutions, including K417T, E484K, and N501Y;^1^ B.1.429 has four mutations, including L452R in the RBD;^4^ the B.1.617.1 variant has 3–7 mutations, including L452R and E484Q in the RBD; B.1.617.2 has ten mutations, including L452R and T478K in the RBD;^5^ and B.1.1.529 has 37 mutations in the S protein, including G339D, S371L, S373P, S375F, K417N, N440K, G446S, S477N, T478K, E484A, Q493R, G496S, Q498R, N501Y and Y505H in the RBD.^6^

The B.1.1.7 variant appears to have minimal impact on the neutralization potency in convalescent and postvaccination sera.^1,3,7–9^ The P.1, B.1.429 and B.1.617.1 variants show moderate neutralization resistance, with a 3–7-fold reduction in the neutralizing antibody (NAb) titers.^1,7,10^ The B.1.351 and B.1.617.2 variants show significantly increased neutralization resistance to convalescent and vaccinated serum samples, as demonstrated by a 7–42-fold reduction in NAb titers.^1,3,7,11^ The B.1.1.529 variant induces convalescent and postvaccination sera to lose most of their neutralizing ability.^12–14^

Consistent with the decline in neutralizing antibodies, the protective efficacy of multiple vaccines is also significantly reduced in areas where the variants are endemic. NVX-CoV2373 achieves an efficacy of 96.4% against COVID-19 caused by the original SARS-CoV-2 in the United Kingdom, whereas its efficacy decreases to 51.0% among HIV-negative trial participants in South Africa, where the majority of the strains belong to the Pangolin lineage B.1.351.^15^ BNT162b2 shows 95% efficacy against infection with the original strain, but its estimated effectiveness is 89.5%, 75.0% and 51.9% against any infection with the B.1.1.7, B.1.351 and B.1.617.2 variants, respectively, in Qatar.^16,17^ The efficacy of Ad26.COV2.S against moderate-to-severe disease is 72.0% in the United States and decreases to 64.0% in South Africa, where 94.5% of patients are infected with the B1.351 variant.^18^ The efficacy of the ChAdOx1 nCoV-19 vaccine was 66.7% before the emergence of the B.1.351 and P.1 variants;^19^ however, this vaccine does not show protection against mild-to-moderate disease due to the B.1.351 variant in South Africa.^20^ Primary immunization with two doses of ChAdOx1 nCoV-19 or BNT162b2 vaccine just provided limited protection against symptomatic disease caused by the B.1.1.529 variant and the vaccine effectiveness dropped to below 10% at 20 or more weeks.^21^ However the booster vaccination substantially increased protection, regardless of which vaccine was used for the booster immunization.^21,22^

Although the current vaccines based on the original strain still appear to prevent severe disease, new vaccines that are effective against SARS-CoV-2 variants as well as the original strain are needed. In this study, we investigated the immunogenicity of the S proteins of the B.1.1.7, B.1.351, P.1, B.1.429, B.1.617.1, B.1.617.2 and B.1.1.529 variants based on the replication-defective human type 5 adenovirus (Ad5) vector. The breadth and magnitude of the immune responses against VOCs obtained with single-dose or prime-boost combined vaccination strategies provide important recommendations for a broader decision-making framework on the vaccine composition.

## Results

### Construction and identification of Ad5-vectored SARS-CoV-2 variant vaccines

**Fig. 1:**
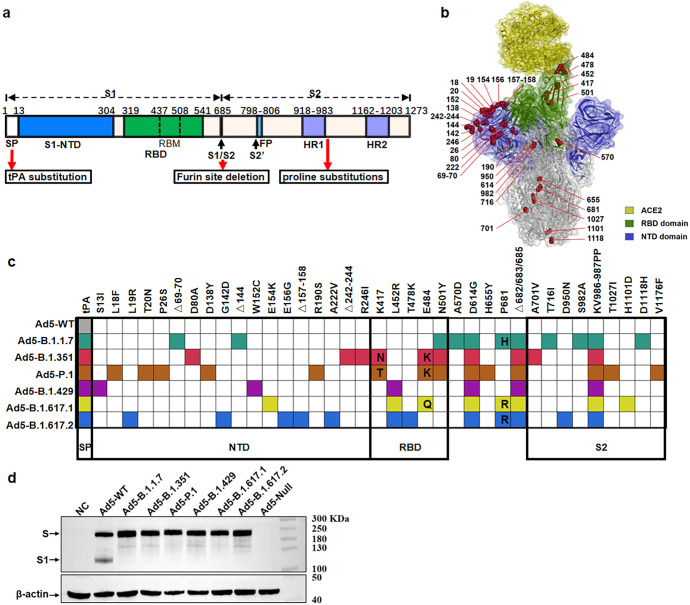
Design of Ad5-vectored SARS-CoV-2 variant vaccines. **a** Schematic organization of the SARS-CoV-2 S protein indicating the domains. The signal peptides were replaced with the tissue plasminogen activator, the furin cleavage site was deleted, and two proline substitutions at residues K986 and V987 were generated. The site mutations found in the S proteins of the B.1.1.7, P.1.351, P.1, B.1.429, B.1.617.1 and B.1.617.2 variants are noted in the S protein structure diagram (**b**, constructed using BIOVIA Discovery Studio Visualizer version 4.5 software) and listed in the table (**c**). **d** The transgene expression levels of Ad5-WT and Ad5-variant vaccines were validated through western blotting after infection in HEK293 cells

To understand the immunogenicity of the S protein of SARS-CoV-2 variants, we prepared a variety of variant vaccines using replication-defective adenovirus type 5 as the model vector. The Ad5 vector expressing the full-length S protein of the wild-type (WT) Wuhan-Hu-1 strain (NC_045512.2) (Ad5-WT) was developed previously^23^ and was used here as an important control. In Ad5-WT, the S gene (aa 14-1273) is codon-optimized, and the signal peptide (aa 1-13) is replaced with the tissue plasminogen activator (tPA). Based on this control, we constructed recombinant Ad5 vectors encoding the S proteins from the B.1.1.7 (Alpha, GISAID, EPI_ISL_718726), B.1.351 (Beta, GISAID, EPI_ISL_1239301), P.1 (Gamma, GISAID, EPI_ISL_804819), B.1.429 (Epsilon, GISAID, EPI_ISL_1032411), B.1.617.1 (Kappa, GISAID, EPI_ISL_1360328) and B.1.617.2 (Delta, GISAID, EPI_ISL_1969244) lineages using a similar method. tPA signal peptide replacement, the furin cleavage site mutation (R682/R683/R685 deletions), and two proline substitutions at the S residues K986 and V987 were made to all S proteins of the variants to produce a more stable and intact S protein (Fig. 1a). The mutation sites in the S proteins of these VOCs are marked three-dimensionally in Fig. 1b and summarized in Fig. 1c. To test the expression level of S antigens, HEK293 cells infected with the above-mentioned Ad5-vectored vaccines were analyzed by western blotting. The results showed that full-length S proteins were expressed in all cells infected with Ad5-vectored variant vaccines, and the expression levels of Ad5-vectored SARS-CoV-2 variant S proteins were quite similar and even slightly higher than the Ad5-WT levels. Moreover, due to deletion of the furin cleavage site in the S protein, cleaved-S1 could only be detected after infection with Ad5-WT but not with the generated variant constructs (Fig. 1d).

### Single-dose vaccination with Ad5-vectored variant S proteins elicits antibodies with strong cross-binding reactivity but distinct neutralizing activity

**Fig. 2:**
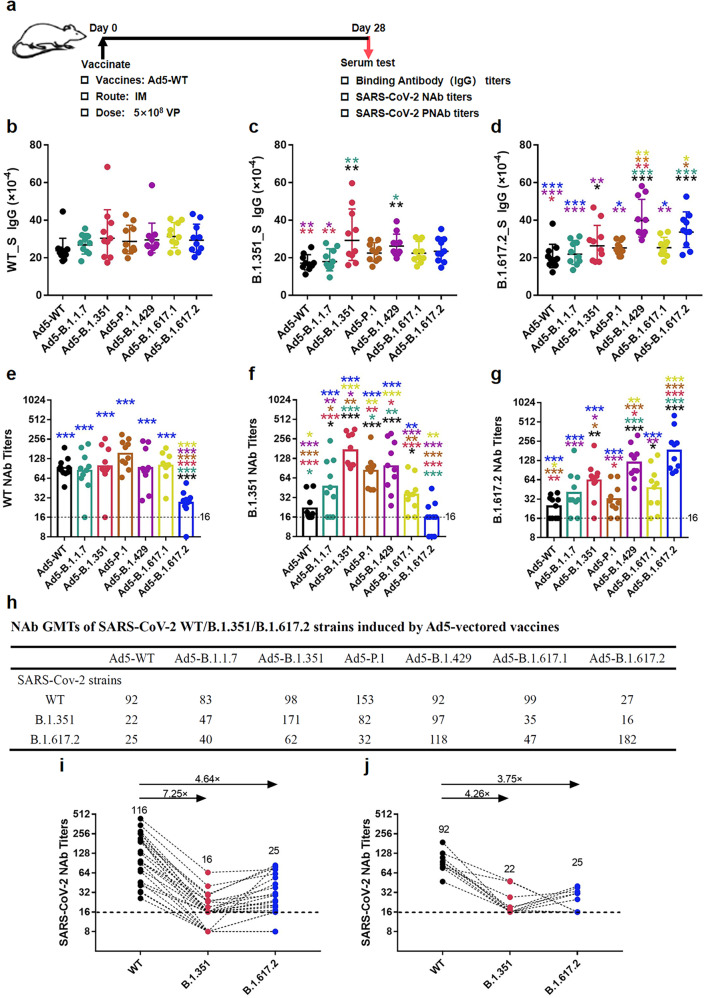
Binding and neutralizing antibodies induced by Ad5-vectored SARS-CoV-2 variant vaccines. BALB/c mice (*n* = 10 per group) received a single intramuscular immunization with Ad5-B.1.1.7, Ad5-B.1.351, Ad5-P.1, Ad5-B.1.429, Ad5-B.1.617.1, B.1.617.2, Ad5-WT or Ad5-null. **a** Immunization schedule of Ad5-vectored SARS-CoV-2 variant vaccines. IgG binding antibodies against S of WT (**b**), B.1.351 (**c**) and B.1.617.2 (**d**) were detected by ELISA at 28 days post-immunization, and the neutralizing antibody titers against WT (**e**) and the B.1.351 (**f**) and B.1.617.2 (**g**) variants were obtained through the microneutralization assay and listed in the table (**h**). The bar graphs show the geometric mean values, and the data of the Ad5-null control group are not shown because all of them were seronegative. The statistical significance was determined by one-way ANOVA with Tukey’s multiple comparisons tests. The NAbs against WT, B.1.351 and B.1.617.2 in serum from 25 convalescent patients (**i**) and Ad5-WT immunized mice (**j**) were used to compare the difference in neutralizing activity between convalescent sera and vaccinated mice sera

To understand the antibody response characteristics to distinct variant strains induced by the Ad5-vectored vaccine in a single-dose immunization regimen, BALB/c mice (*n* = 10 per group) were immunized intramuscularly with 5 × 10^8^ viral particles (VPs) of Ad5-vectored variant S to evaluate their immunogenicity. Twenty-eight days after immunization, sera samples were tested for binding to the S proteins of different variants (Fig. 2a). No significant difference in the IgG antibody responses to WT_S was observed among the different groups (Fig. 2b). As expected, the highest levels of anti-B.1.351_S IgG antibodies were induced by Ad5-B.1.351, followed by Ad5-B.1.429, and the levels induced by both of these variants were significantly higher than those induced by Ad5-WT and Ad5-B.1.1.7 (Fig. 2c). Similarly, anti-B.1.617.2_S IgG antibodies were strongly elicited by Ad5-B.1.429 and Ad5-B.1.617.2, and the resulting levels were significantly higher than those induced by Ad5-WT, Ad5-B.1.1.7, Ad5-P.1 and Ad5-B.1.617.1 (Fig. 2d).

Of note, although the highest levels of self-matched anti-S IgG antibodies were generated by Ad5-B.1.351 and Ad5-B.1.617.2, the fold changes in the geometric mean titers (GMTs) did not exceed a twofold change among the different vaccination groups. These results indicated that the IgG antibodies induced by SARS-CoV-2 variant S proteins exhibited general cross-binding abilities.

The NAbs induced by the Ad5-vectored variant S proteins were further investigated using the live virus neutralization method, and the results revealed no significant difference in the neutralizing abilities of sera from most of the Ad5-vectored variant S-immunized mice against the SARS-CoV-2-WT strain, with the exception of the sera from mice immunized with Ad5-B.1.617.2 (Fig. 2e). The highest neutralizing capacity of sera against B.1.351 was detected in the mice immunized with Ad5-B.1.351, and this level was slightly higher than the results obtained with Ad5-B.1.429 and Ad5-P.1; however, significantly reduced NAb titers against B.1.351 were detected in the mice immunized with Ad5-WT and Ad5-B.1.617.2 (Fig. 2f). Similarly, the highest levels of NAbs against B.1.617.2 were induced by Ad5-B.1.617.2, followed by Ad5-B.1.429, and both of these levels were significantly higher than those induced by Ad5-WT, Ad5-B.1.1.7 and Ad5-P.1 (Fig. 2g). The NAb GMTs in each group were listed in Fig. 2h. Consistent with the anti-S IgG antibody results, the highest levels of self-matched NAbs were generated by Ad5-WT, Ad5-B.1.351 and Ad5-B.1.617.2. NAbs showed higher variant specificity than the binding IgG antibodies for different variants. NAbs from the Ad5-WT and Ad5-B.1.1.7 against B.1.351 and B.1.617.2 were significantly decreased compared to those against the WT strain. In addition, Ad5-B.1.351 showed a stronger cross-neutralizing effect compared with Ad5-B.1.617.2.

We questioned whether the neutralizing capabilities of immunized sera from mice coincided with natural infections; therefore, the NAb titers of 25 serum samples collected in September 2020 from patients with COVID-19 infected with the SARS-CoV-2 original strain who were convalescent for more than 6 months were detected (Fig. 2i). The NAb GMTs against the B.1.351 and B.1.617.2 variants were significantly reduced from 116 against the WT to 16 and 25, resulting in fold changes of 7.25 and 4.64, respectively. Correspondingly, the NAb GMTs against the WT, B.1.351 and B.1.617.2 strains in sera from mice 4 weeks after immunization with Ad5-WT were 92, 22, and 25, respectively (Fig. 2j). The results illustrated that the NAb response from mice immunized with Ad5-vectored variant S proteins could clinically reflect the NAb response from patients with COVID-19 to a certain extent.

### Single-dose vaccination exerts optimal self-matched neutralizing effects, and in particular, the Ad5-B.1.351 vaccine elicits broad-spectrum cross-neutralizing antibodies

To measure the neutralizing response against more types of SARS-CoV-2 variants, we constructed HIV-1-based virions carrying a luciferase reporter that were pseudotyped with the S protein from SARS-CoV-2 variants. A significant linear correlation was found between virus dilution and luciferase activity (Supplementary Fig. S1). The pseudovirus neutralizing antibody (PNAb) GMTs of serum from Ad5-WT-immunized mice against B.1.351 and B.1.617.2 were significantly lower (205 and 282, respectively) than those against the WT strain (708), which corresponded to 3.42- and 2.51-fold reductions (Supplementary Fig. S2), and these results were consistent with live virus NAb results (Fig. 2j).

**Fig. 3:**
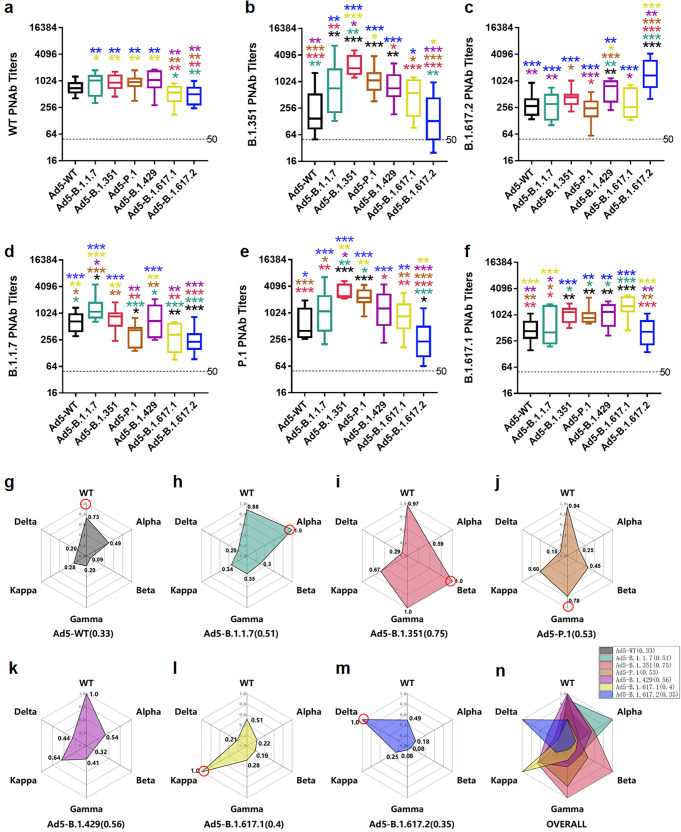
Pseudovirus neutralizing antibodies induced by Ad5-vectored SARS-CoV-2 variant vaccines. Sera of mice were collected at 4 weeks post-immunization. The PNAb titers of the different variant vaccine groups were obtained using HIV backbone-derived pseudovirus bearing S proteins of the WT strain (**a**) and the B.1.351 (**b**), B.1.617.2 (**c**), B.1.1.7 (**d**), P.1 (**e**) and B.1.617.1 (**f**) variants. The statistical significance among the different groups was determined by one-way ANOVA with Tukey’s multiple comparisons tests. The PNAb GMTs to a certain type of pseudovirus were normalized by dividing by the highest value of PNAb GMT to the matched pseudovirus in all vaccine groups, and the broad-spectrum neutralization abilities of Ad5-WT (**g**), Ad5-B.1.1.7 (**h**), Ad5-B.1.351 (**i**), Ad5-P.1 (**j**), Ad5-B.1.429 (**k**), Ad5-B.1.617.1 (**l**), and B.1.617.2 (**m**) and the overall picture (**n**) are shown. The self-matched PNAbs are noted by red circles

Through PNAb assays, an optimal self-matched neutralizing response was also observed in response to corresponding Ad5-vectored variant S proteins at 28 days post-single-dose vaccination, and cross-neutralization also occurred to different extents. The PNAbs against SARS-CoV-2-WT were induced nearly indiscriminately by Ad5-WT, Ad5-B.1.1.7, Ad5-B.1.351, Ad5-P.1, and Ad5-B.1.429 but not by Ad5-B.1.617.2 and Ad5-B.1.617.1 (Fig. 3a). Ad5-B.1.351, Ad5-P.1, Ad5-B.1.1.7 and Ad5-B.1.429 induced higher PNAb titers against B.1.351 than those of Ad5-WT and Ad5-B.1.617.2 (Fig. 3b). Ad5-B.1.617.2 and Ad5-B.1.429 generated higher PNAb titers against B.1.617.2 than those of Ad5-WT, Ad5-B.1.1.7 and Ad5-P.1 (Fig. 3c). These results showed remarkable consistency with results in live virus NAbs (Fig. 2e–g). In addition, the Ad5-B.1.1.7, Ad5-WT, Ad5-B.1.351, and Ad5-B.1.429 induced higher PNAb titers against B.1.1.7 than those of the Ad5-P.1, Ad5-B.1.617.1, and Ad5-B.1.617.2 (Fig. 3d). Ad5-P.1, Ad5-B.1.351, and Ad5-B.1.429 elicited higher PNAb titers against P.1 and the lowest PNAb titer was from Ad5-B.1.617.2 (Fig. 3e). Finally, Ad5-B.1.617.1, Ad5-B.1.351, Ad5-P.1 and Ad5-B.1.429 elicited higher PNAb titers against B.1.617.1 and the lowest PNAb titer was from Ad5-B.1.617.2 (Fig. 3f).

Subsequently, we aimed to better elucidate the broad-spectrum NAb responses against different variants elicited by a single vaccine. The PNAb GMTs to a certain type of pseudovirus were normalized by dividing by the highest PNAb GMT to the matched pseudovirus in all vaccine groups, and the PNAb GMT of each Ad5-vectored variant S was summarized by a radar chart (Fig. 3g–n). The size of the shadow represents the neutralizing cross-activity. Obviously, the Ad5-WT vaccine lost a large portion of resistance to these variants (Fig. 3g). Of particular concern is Ad5-B.1.351, which exhibited broad-spectrum cross-neutralizing protection against diverse variants, particularly against the WT, Gamma and Kappa variants, but not the Delta variant (Fig. 3i). In addition, Ad5-B.1.1.7, Ad5-P.1 and Ad5-1.429 harbored moderate cross-neutralizing ability but could provide good protection against the WT strain (Fig. 3h, j, k). In contrast, the cross-NAb responses induced by Ad5-B.1.617.1 and Ad5-B.1.617.2 were rather weak, and the neutralizing spectrum of them was relatively centered and exhibited only superior neutralizing effects on their own (Fig. 3l, m). Overall, these results demonstrated that each variant S protein induced the best self-matched NAbs after a single vaccination and B.1.351 S protein elicited the broadest cross-neutralizing activity (Fig. 3n).

### Ad5-B.1.617.2- or Ad5-B.1.429-boost vaccination exhibits optimal neutralization against diverse VOCs after Ad5-WT-prime vaccination

**Fig. 4:**
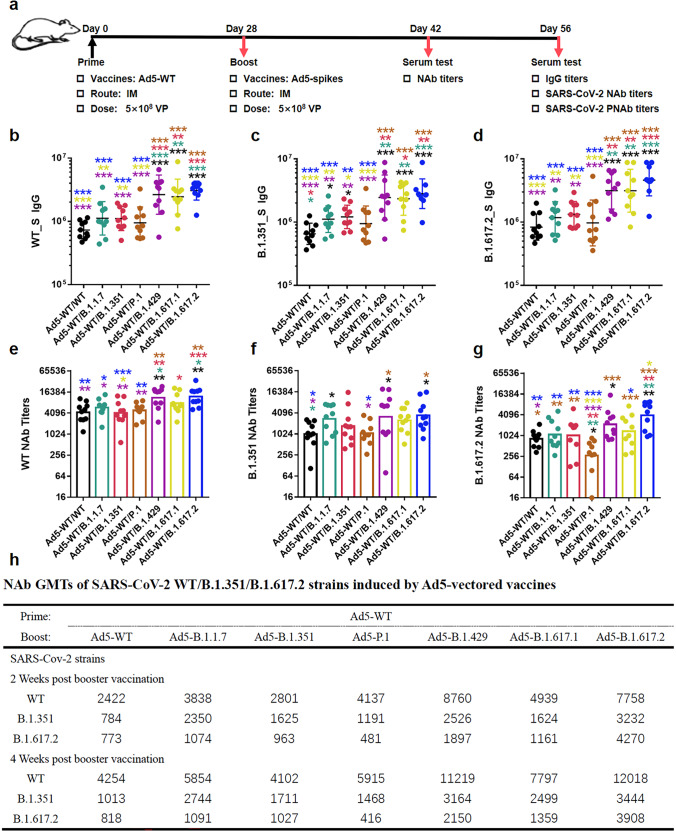
Binding and neutralizing antibodies induced by Ad5-WT-prime and variant vaccine-booster vaccination. BALB/c mice (*n* = 10 per group) were primed with Ad5-WT and boosted with Ad5-B.1.1.7, Ad5-B.1.351, Ad5-P.1, Ad5-B.1.429, Ad5-B.1.617.1, B.1.617.2, Ad5-WT or Ad5-null. **a** Immunization schedule of Ad5-vectored SARS-CoV-2 variant vaccines. IgG-binding antibodies and NAbs against WT (**b**, **e**) and the B.1.351 (**c**, **f**) and B.1.617.2 (**d**, **g**) variants were obtained at 28 days post-booster immunization. The GMTs of NAb at 14 or 28 days post-booster immunization are listed in the table (**h**). The bar graphs show the GMT values, and the data of the Ad5-null control group are not shown because all of them were seronegative. The statistical significance was determined by one-way ANOVA with Tukey’s multiple comparisons tests

Considering that the WT COVID-19 vaccines have been widely used, the Ad5-WT-prime and Ad5-variant-boost vaccinations were tested. BALB/c mice (*n* = 10 per group) were intramuscularly primed with 5 × 10^8^ VPs of recombinant Ad5-WT and boosted with the same dose of Ad5-vector S proteins after 4 weeks (Fig. 4a). The binding IgG antibody, live virus NAb and PNAb were measured on 28 days after booster immunization. Ad5-B.1.617.2, Ad5-B.1.429 or Ad5-B.1.617.1 induced higher anti-S IgG antibodies against WT, B.1.351, and B.1.617.2 S proteins than those of Ad5-WT, Ad5-B.1.1.7, Ad5-B.1.351 or Ad5-P.1 (Fig. 4b–d). Remarkably, single-dose vaccination with Ad5-B.1.351 was statistically superior for anti-B.1.351-S IgG (Fig. 2c), but no additional advantage was observed with Ad5-WT/Ad5-B.1.351 prime-boost vaccination (Fig. 4c).

Similarly, the optimal neutralizing effect against WT, B.1.351 and B.1.617.2 strains was also observed when Ad5-B.1.617.2 or Ad5-B.1.429 was administered as the boost vaccine, followed by Ad5-B.1.617.1 (Fig. 4e–g). Notably, Ad5-WT/Ad5-B.1.617.2 vaccination significantly augmented the neutralizing response against WT and B.1.351 strains compared with that obtained after single-dose vaccination with Ad5-B.1.617.2 alone.

In addition, a slight increase in NAb GMTs was observed from day 14 to day 28 post-boost (Fig. 4h). Twenty-eight days after boosting, the levels of NAbs against the WT, B.1.351 and B.1.617.2 variants were at least 13 times and up to 45 times higher than those pre-boost (NAbs against the WT, B.1.351 and B.1.617.2 strains were 92, 22, 25, respectively, from mice immunized with Ad5-WT 4 weeks after single immunization). In addition, across all seven groups, the NAb GMTs against the WT strain were ~2.1–14.2 times higher than those against the B.1.351 and B.1.617.2 variants, indicating the superiority of the prime-boost vaccination in terms of NAbs against WT strain.

### The convergent antibody responses to VOCs with prime-boost vaccination determine the cross-neutralizing activity

**Fig. 5:**
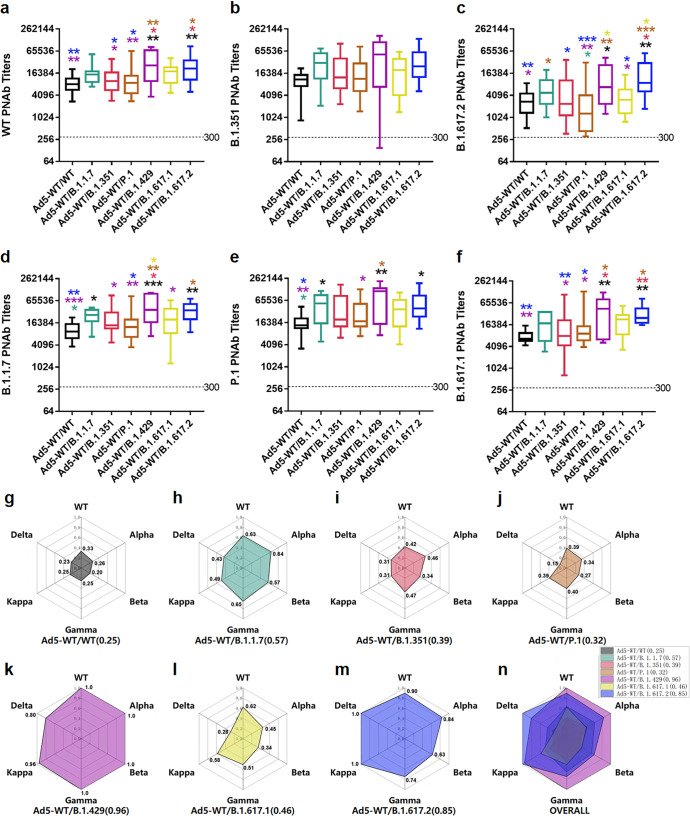
Pseudovirus neutralizing antibodies induced by Ad5-WT-prime and variant vaccine-booster vaccination. Sera of mice were collected at 28 days post-booster immunization. The PNAb titers against the WT strain (**a**) and the B.1.351 (**b**), B.1.617.2 (**c**), B.1.1.7 (**d**), P.1 (**e**) and B.1.617.1 (**f**) variants in different variant vaccine groups were obtained. The statistical significance among different groups was determined by one-way ANOVA with Tukey’s multiple comparisons tests. The PNAb GMTs to a certain type of pseudovirus were normalized by dividing by the highest value of PNAb GMT to the matched pseudovirus in all booster groups. The broad-spectrum neutralization abilities of the vaccine strategy involving boosting with Ad5-WT (**g**), Ad5-B.1.1.7 (**h**), Ad5-B.1.351 (**i**), Ad5-P.1 (**j**), Ad5-B.1.429 (**k**), Ad5-B.1.617.1 (**l**), and B.1.617.2 (**m**) and the overall picture (**n**) are shown

We then measured the neutralizing response 4 weeks post-boost vaccination through pseudovirus neutralization assays. The PNAb titers obtained with single-dose or prime-boost vaccination regimens showed high correlations with the live virus NAb titers for the WT, B.1.351 and B.1.617.2 strains (*R*^2^ = 0.854, 0.911 and 0.731, respectively, calculated by Pearson correlation analysis (*p* < 0.0001), Supplementary Fig. S3). The overall level of neutralizing activity against pseudotyped WT and each VOC was generally high after the heterologous administration of Ad5-B.1.617.2 or Ad5-B.1.429 to Ad5-WT-primed cohorts and was significantly superior to those of Ad5-WT, Ad5-B.1.351, and Ad5-P.1 boosters (Fig. 5a–f). Remarkably, the relative levels of PNAbs among the different booster groups remained similar regardless of the type of pseudovirus, that is, Ad5-B.1.429 ≈ Ad5-B.1.617.2 > Ad5-B.1.617.1 ≈ Ad5-B.1.1.7 > Ad5-B.1.351 ≈ Ad5-P.1 ≈ Ad5-WT. The results also indicated that prime-boost immunization induced more broad-spectrum cross-neutralizing activity against diverse VOCs than single vaccination.

Subsequently, the PNAb GMTs were also outlined by radar charts (Fig. 5g–n). Clearly, the widest spectrum of pseudovirus NAb responses was obtained with Ad5-B.1.429 and Ad5-B.1.617.2 boosters, followed by Ad5-B.1.617.1 and Ad5-B.1.1.7 boosters and then by Ad5-B.1.351, Ad5-P.1 and Ad5-WT boosters. Moreover, the “radar” shape obtained with the prime-boost vaccination strategy was closer to a round or oval rather than the sharp-edged polygon shape obtained with single-dose vaccination (Fig. 3g–n), which suggested that prime-boost vaccination induced convergent cross-reactive antibody responses to VOCs.

Furthermore, to compare the neutralization activity of the induced convergent cross-reactive antibody responses against different variants, the PNAb titers were normalized by dividing by those against the WT strain to make the PNAb value against WT pseudovirus in each vaccination group equal to 1 (Supplementary Fig. S4). Apparently, the protective immunity spectrum induced by distinct booster immunization was particularly consistent: the highest neutralization potency was found against P.1, the lowest potency was observed against B.1.617.2, and moderate and comparable neutralizing potency was found against WT and the B.1.1.7, B.1.351, and B.1.617.1 variants (Supplementary Fig. S4b). In contrast, the variation in the NAb spectrum against different pseudoviruses was more obvious with the single-dose vaccination strategy (Supplementary Fig. S4a). It could be emphasized that boost immunization with variants based on Ad5-WT prime vaccination would induce consistent convergent antibody responses, which provide strong protection against both WT and variant strains of SARS-CoV-2.

### Ad5-vectored S protein of Omicron showed highly immunogenic against self-matched Omicron variants

Omicron (B.1.1.529) is known to escape the majority of existing SARS-CoV-2 NAbs elicited by original COVID-19 vaccines. Herein, according to the sequence of the B.1.1.529 variant (GISAID, EPI_ISL_6640917, also defined as the BA.1 variant), which was responsible for the first outbreak in South Africa, we constructed the Ad5-based Omicron vaccine Ad5-B.1.1.529 using a similar approach to that used for the other variant vaccines described above. Ad5-B.1.1.529 could express S protein at levels comparable to those obtained after infection with Ad5-WT, Ad5-B.1.351 or Ad5-B.1.1617.2 in HEK293 cells (Fig. S5). To test the immunogenicity of Ad5-B.1.1.529 obtained with the single-dose or prime-boost immunization strategies used for the other variant vaccines described before, BALB/c mice were immunized with the Ad5-B.1.1.529 at a dose of 5 × 10^8^ VPs, and another group of mice was primed with Ad5-WT and boosted with Ad5-B.1.1.529 at 4-week intervals. Sera were collected 28 days after the last immunization and tested for specific antibodies against diverse SARS-CoV-2 variants.

**Fig. 6:**
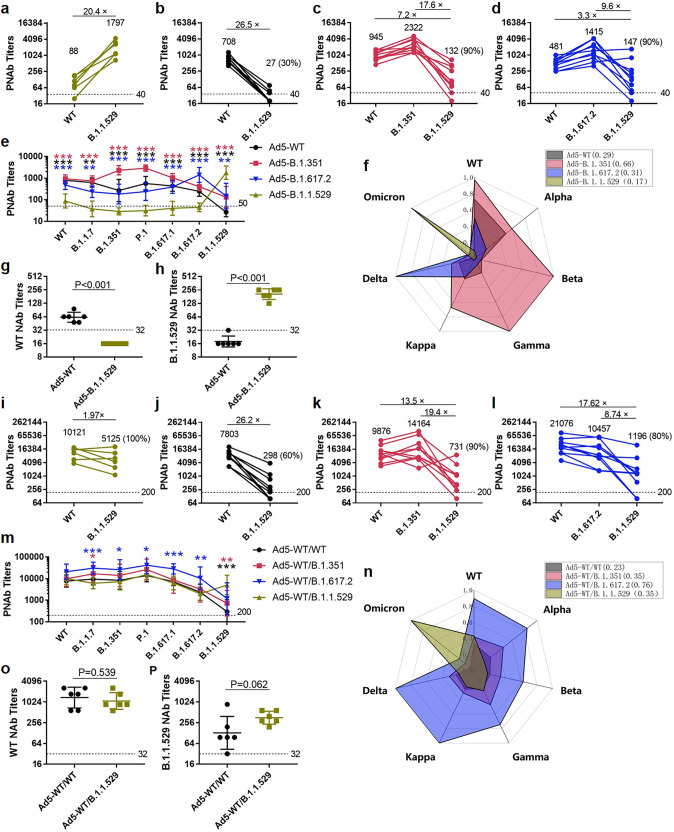
Self-matched neutralizing activities elicited by the Ad5-vectored spike protein of Omicron. The PNAbs against WT, Omicron or self-matched variants at 28 days after immunization with a single-dose of Ad5-B.1.1.529, Ad5-WT, Ad5-B.1.351 and Ad5-B.1.617.2 are shown (**a**–**d**), and the PNAbs to distinct variants are drawn in the line chart (**e**) and normalized in the radar chart (**f**). The NAbs against SARS-CoV-2 WT and Omicron strain are shown (**g**, **h**). Mice were primed with Ad5-WT and were boosted with Ad5-vectored S proteins 28 days after first vaccination. The PNAbs against WT, Omicron or a self-matched variant after 28 days boosted with the Ad5-B.1.1.529, Ad5-WT, Ad5-B.1.351 and Ad5-B.1.617.2 are shown (**i**–**l**), and PNAbs to distinct variants in four prime-boost vaccination groups are drawn in the line chart (**m**) and normalized in the radar chart (**n**). The NAbs against SARS-CoV-2 WT and Omicron strain in Ad5-WT- and Ad5-B.1.1.529-boosted groups are shown (**o**, **p**)

With the single-dose immunization regimen, Ad5-B.1.1.529 induced higher serum IgG titers than Ad5-WT, especially against the spike of Omicron variant (Supplementary Fig. S6a,b). For PNAb titers, high PNAb titers against Omicron variant, but significantly low PNAb titers against WT strain (Fig. 6a) were elicited by Ad5-B.1.1.529. Similarly, Ad5-WT, Ad5-B.1.351, Ad5-B.1.617.2, Ad5-B.1.429, Ad5-B.1.1.7, Ad5-P.1 and Ad5-B.1.617.1 induced lower PNAb titers against Omicron than those against self-match variants (Fig. 6b–d and Supplementary Fig. S7a–d). In addition, the cross-neutralizing capacity against the B.1.1.7, B.1.351, P.1, B.1.617.1 and B.1.617.2 variants induced by immunization with Ad5-B.1.1.529 alone was generally lower than those exerted by Ad5-B.1.351, Ad5-B.1.617.2 or Ad5-WT (Fig. 6e). The radar chart shows that the Ad5-B.1.1.529 has the strongest antigenic uniqueness (Fig. 6f). The results obtained using live virus confirmed that the serum NAbs induced by Ad5-B.1.1.529 and Ad5-WT had little cross-neutralizing reactivity (Fig. 6g, h). B.1.1.529 S protein only stimulated self-matched neutralizing antibodies, while other variant S proteins could induce significantly lower cross-neutralizing antibodies against B.1.1.529.

With the prime-boost immunization regimen, Ad5-B.1.1.529-boosted group induced similar level of serum IgG against spike of WT and slightly higher serum IgG against spike of Omicron variant than Ad5-WT-boosted group (Supplementary Fig. S6c, d). The mice primed with Ad5-WT and boosted with distinct Ad5-vectered S proteins, particularly the mice in the Ad5-B.1.1.529, Ad5-B.1.617.2 and Ad5-B.1.351 boosted groups, showed markedly higher PNAbs against Omicron than the mice immunized with a single-dose of Ad5-WT (the PNAb GMT to Omicron is 27) (Fig. 6i–l and Supplementary Fig. S7e–h). In addition, PNAb titers against Omicron in the Ad5-B.1.617.2- and Ad5-B.1.351-boosted groups (GMT = 1196 and 731, respectively) were similar to those obtained 28 days after immunization with Ad5-B.1.1.529 alone (GMT = 1797). The highest PNAbs against the Omicron variant were obtained after boosting with Ad5-B.1.1.529; however, the cross-neutralizing activity against the WT and B.1.1.7, B.1.351, P.1, B.1.617.1 and B.1.617.2 variants was similar to that obtained with boosting with Ad5-WT and lower than that obtained with boosting with Ad5-B.1.617.2 or Ad5-B.1.351 (Fig. 6m, n). Live virus NAb results also proved that similar level of neutralizing capacity against WT strain (Fig. 6o) and higher level of neutralizing capacity against Omicron variant (Fig. 6p) were induced by Ad5-B.1.1.529 boosters compared with Ad5-WT boosters.

## Discussion

In September 2021, the WHO established the Technical Advisory Group on COVID-19 Vaccine Composition to assess the strain composition of COVID-19 vaccines and encourages vaccine developers to gather data on a small scale on the breadth and magnitude of immune responses obtained with monovalent and multivalent vaccines against VOCs. In our study, we constructed and identified diverse Ad5-vectored SARS-CoV-2 variant S proteins, including Ad5-B.1.1.7, Ad5-B.1.351, Ad5-P.1, Ad5-B.1.429, Ad5-B.1.617.1, Ad5-B.1.617.2 and Ad5-B.1.1.529, and explored the immunogenicity against distinct VOCs obtained through single-dose and boost vaccination based on Ad5-WT priming vaccination strategies.

The evaluation assays used in this study for the detection of humoral antibody responses included anti-S IgG assessment, live virus neutralizing assay, and pseudovirus-based neutralizing assay. These methods exhibited good consistency, and more importantly, the neutralizing activities of immune sera from mice coincided well with those observed with natural infection. On the one hand, single-dose vaccination with SARS-CoV-2 variant vaccines could effectively stimulate specific antibody responses with strong cross-binding activity but distinct neutralizing activity. Variant S proteins elicited the optimal self-matched neutralizing activities and almost indistinguishable neutralizing activities against the WT strain after a single vaccination although there were only a few amino acid differences among these variants. In particular, the Ad5-B.1.351 exhibited the most broad-spectrum cross-neutralizing response against a wide range of SARS-CoV-2 variants.

On the other hand, in the prime-boost vaccination regimen, Ad5-WT was designated as the prime dose based on the currently widely used WT COVID-19 vaccine. When boosted by different SARS-CoV-2 variant vaccines, the pattern of the NAb response against diverse SARS-CoV-2 variants was strikingly similar, which indicated that distinct boosters elicited convergent antibody responses, and this finding further emphasizes the general characteristics shared by boosted vaccines. Convergent antibody responses have been reported previously, and unrelated individuals can produce genetically similar clones of antibodies.^24,25^ Herein, we also observed convergent antibody responses induced by distinct boosters. In addition, we discovered that these convergent NAbs showed broad-spectrum cross-neutralizing protection against diverse VOCs: the highest neutralization potency was observed against P.1, the lowest potency was found against B.1.617.2, and moderate and comparable neutralizing potential was found against WT and the B.1.1.7, B.1.351, and B.1.617.1 variants. In particular, sequential doses of Ad5-B.1.617.2 and Ad5-B.1.429 exhibited the optimal neutralizing capacity against VOCs, followed by Ad5-B.1.617.1. Because the RBD is crucial for interaction with NAbs,^26^ the RBDs of B.1.617.2, B.1.429 and B.1.617.1 harbor a specific L452R mutation, which implies that L452R is the crucial mutation influencing the convergent cross-reactive antibody response.

The B.1.1.7 variant contains one substitution (N501Y) in the RBD, which mainly increases the affinity of the S protein for ACE2 receptors and thus enhances viral attachment and transmissibility^27^ but mostly preserves neutralizing activity.^28^ Our results also showed that single-dose vaccination with Ad5-WT or Ad5-B.1.1.7 induced superior and comparable neutralizing activity against B.1.1.7 and WT viruses. Thus, Ad5-B.1.1.7 and Ad5-WT vaccination could provide potent protection against both WT and Alpha variants, with an over 30-fold increase after boosting, but showed the lowest cross-neutralizing activities against the other variants.

The B.1.351 and P.1 variants contain three substitutions (K417, E484K and N501Y) in the RBD. Among these, the K417N and E484K mutations are known to attenuate the neutralizing activity of convalescent sera,^29^ leading to loss of plasma neutralizing activity against Beta and Gamma variants after immunization with the WT COVID-19 vaccine. Notably, single-dose vaccination with Ad5-B.1.351 or Ad5-P.1 could elicit robust NAb responses against WT and the B.1.351, P.1, and B.1.617.1 variants. In particular, single-dose Ad5-B.1.351 vaccination elicited highly broad cross-neutralizing activities; however, no further advantages were observed with Ad5-WT/Ad5-B.1.351 prime-boost vaccination, which induced a level similar to that obtained after boosting with Ad5-WT and Ad5-B.1.1.7.

The B.1.617.1, B.1.617.2, and B.1.429 variants harbor the S substitution L452R, which reduces susceptibility to convalescent and vaccine-elicited sera and mAbs^30^ but shows considerable differences in immunogenicity after single vaccination. Ad5-B.1.429 triggered moderate neutralizing potential against diverse variants, whereas both Ad5-B.1.617.1 (+E484Q) and Ad5-B.1.617.2 (+T478K) induced prominent self-matched neutralizing effects. Priming with Ad5-WT and boosting with Ad5-B.1.617.2, Ad5-B.1.429, and Ad5-B.1.617.1 induced robust binding and NAbs against a wide range of VOCs, particularly Ad5-B.1.617.2 and Ad5-B.1.429.

The Omicron variant has dominated the SARS-CoV-2 variants around the world due to its enhanced transmissibility and increased antibody escape. Omicron shows a marked degree of mutation, harboring up to ten mutations in the receptor-binding motif, and no more than two mutations (only in four sites; L452, T478, E484, and N501) overlap with those in each previous SARS-CoV-2 variant. As reported previously, the neutralization of Omicron is greatly reduced with most vaccines.^6^ In our study, the vaccine based on the S protein of the B.1.1.529 variant can only stimulate self-matched NAbs, and significantly lower cross-NAb titers against B.1.1.529 could be induced by other variant vaccines, including Ad5-WT, Ad5-B.1.351, Ad5-B.1.617.2, Ad5-B.1.429, Ad5-B.1.1.7, Ad5-P.1 and Ad5-B.1.617.1, with the single-dose vaccination strategy. However, with the prime-boost immunization strategy, markedly enhanced cross-neutralizing responses against Omicron were observed with all boosters, particularly with the self-matched Ad5-B.1.1.529 booster, followed by the Ad5-B.1.617.2 and Ad5-B.1.351 boosters. The results suggest that the use of the SARS-CoV-2 vaccine based on the prototype strain for booster immunization can also enhance neutralization resistance to the Omicron variant, but the use of self-matched Omicron vaccines is more effective.

As mentioned above, we observed that prime-boost immunization tended to elicit a class of convergent cross-reactive antibodies. However, the Omicron variant had high potential to evade the neutralizing ability of this type of convergent antibody. Previous studies have revealed that based on sequence alignment differences and gaps, the Omicron variant is phylogenetically distant from other variants and produces a new monophyletic clade.^31^ Our results further ensured that the Omicron variant represented a new emergent form that exhibits significantly different immunogenicity from the Alpha, Beta, Gamma, Epsilon, Kappa and Delta variants, and indicate that the development of updated vaccines against Omicron or other variants originating from Omicron is a matter of great urgency in years to come.

There are some limitations in this work. The neutralizing activities against B.1.1.7, B.1.617.1 and P.1 variant strains were only tested by the pseudovirus-based method because these live variant viruses were not available in our laboratory. The PNAb titers measured here could represent the relative level of live virus neutralization antibody titers for these variants, which could also provide a valuable reference view of cross-reactivity. In addition, our study only provides immunogenicity data induced by Ad5-vectored spike proteins of emerging SARS-CoV-2 VOCs, not in vivo protective effects.

Taken together, our findings demonstrated the antibody response characteristics for single-dose or prime-boost vaccination induced by the S proteins of emerging SARS-CoV-2 VOCs, including the Alpha, Beta, Gamma, Epsilon, Kappa, Delta, and Omicron variants. The heterologous administration of Ad5-B.1.617.2 and Ad5-B.1.429 to Ad5-WT-primed mice resulted in superior antibody responses against most VOCs and Ad5-B.1.1.529 booster elicit the optimal specific NAb response to Omicron and almost similar level of NAbs to other VOCs as Ad5-WT. The results provide an important reference regarding immunogenicity for the current complex situation in which most of the general population has been immunized with the WT vaccine and new VOC are continuously emerging.

## Materials and methods

### Cell culture

HEK293 and Vero E6 cells were obtained from the ATCC. ACE2-293T cells were constructed by stably transfecting the human angiotensin-converting enzyme 2 (ACE2) gene into HEK293T cells under puromycin pressure in our lab. All cells were cultured at 37 °C in a 5% CO_2_ incubator and maintained in Dulbecco’s modified Eagle’s medium (Thermo Scientific, USA) supplemented with 10% fetal bovine serum (Thermo Scientific, USA), penicillin (100 units/ml) and streptomycin (100 μg/ml). Furthermore, no mycoplasma was contained in all cell lines, which had been tested recently.

### SARS-CoV-2 viruses

SARS-CoV-2/human/CHN/Beijing_IME-BJ01/2020 and the Beta (ACC_20SF18530/Guangdong strain), Delta and Omicron variants were isolated from patients and propagated in Vero E6 cells. The virus titers were determined by a standard plaque assay using Vero E6 cells, and virus stocks were stored in aliquots at a temperature below −70 °C.

### Human serum samples

Twenty-five human serum samples were collected on September 2020 from convalescent patients with COVID-19 at Wuhan. All patients were infected with the SARS-CoV-2 WT strain and had recovered for more than half a year. The serum was stored at −70 °C. The experiment involving human serum was performed in accordance with the Declaration of Helsinki and had been approved by institutional ethics committee.

### Construction of Ad5-based variant vaccines

Ad5-WT encoding the S protein of the WT Wuhan-Hu-1 strain (NC_045512.2) was previously constructed and named Ad5-nCoV.^23^ The WT S gene (14-1273 aa) was codon-optimized, and the signal peptides (aa 1-13) were replaced by the tPA. For other Ad5-based variant vaccines, the tPA-substituted and codon-optimized S gene with the furin cleavage site mutation (R682/R683/R685 deletion) and two proline substitutions at residues K986 and V987 were synthesized and cloned into the shuttle plasmid pDC316 of the AdMax adenovirus system (Microbix Biosystem, Canada). After sequencing identification, the shuttle plasmids with the target gene were cotransfected into HEK293 cells with the backbone plasmid (pBHGloxΔE1, 3Cre) using the TurboFect transfection reagent (Thermo Scientific, USA) according to the manufacturer’s instructions. The transfected cells were passaged when they were overgrown and collected until Ad-related cytopathic effects were observed. The cells were lysed through three freeze–thaw cycles to release the recombinant viruses. The recombinant adenoviruses were confirmed by target gene sequencing, amplified by serial passage into HEK293 cells, and purified by ion-exchange and size-exclusion chromatography. The number of total VPs was measured by ultraviolet spectrophotometer analysis (one OD_260_ was equal to ~1.1 × 10^12^ VPs), and the infectious units were titrated on HEK293 cells using an Adeno-X™ Rapid Titer Kit (Clontech, USA) following the manufacturer’s instructions.

### Western blotting

HEK293 cells were seeded into six-well cell culture clusters and transiently transfected with the same amount of Ad5-WT or Ad5-based variant vaccines at MOI = 1. At 24 h posttransfection, the culture supernatant was discarded, and the cells were rinsed and lysed with 150 μl of RIPA Lysis and Extraction Buffer (Thermo Scientific, USA). Then, the lysate was centrifuged and mixed with LDS buffer (Thermo Scientific, USA). The samples were run on a 4–20% PAGE gel (GenScript, China) and transferred to a NC membrane (Bio-Rad, China). After that, Tris-HCl with Tween 20 (0.1%) and skim milk (5%) was added to block the membrane for about 1 h, a SARS-CoV-2 (2019-nCoV) spike antibody (Sino Biological, China) was used to incubate the membrane with a 1:2000 dilution. After washes, goat anti-rabbit IgG (with HRP, Cell Signaling Technology) was used to bind to the primary antibody. After washes again, ECL Substrate (Thermo Scientific, USA) was used to develop the membrane and images were obtained using ChemiScope system (Clinx Science, China). Besides, β-actin expression levels were obtained using similar method with a specific β-actin antibody (with HRP, Abcam, UK).

### Animal experiments

Animal experiments were performed according to the guidelines of the Institutional Experimental Animal Welfare and Ethics Committee. BALB/c mice aged 42–56 days were purchased from Vital River Laboratories (Beijing, China) and used for all experimental groups. With the single-dose vaccination strategy, 80 mice were randomly divided into eight groups and immunized intramuscularly with 5 × 10^8^ VP of Ad5-WT, Ad5-B.1.1.7, Ad5-B.1.351, Ad5-P.1, Ad5-B.1.429, Ad5-B.1.617.1, Ad5-B.1.617.2 or Ad5-null (control, no transgene expression) at day 0. Sera were collected at 4 weeks post-immunization for S-specific IgG ELISAs, pseudovirus neutralization assays and live SARS-CoV-2 microneutralization assays. With the prime-boost combined vaccination strategy, 80 mice were primed with Ad5-WT at day 0 and boosted with Ad5-vectored WT or variant vaccines at day 28, and the antibody responses were detected at days 42 and 56. For the assessment of immunogenicity against Omicron, two other groups (*n* = 6) were added: one was immunized intramuscularly with 5 × 10^8^ VPs of Ad5-B.1.1.529, and the other was primed with Ad5-WT and boosted with Ad5-B.1.1.529 at 4-week intervals.

### ELISA

The S proteins for ELISAs were obtained from ACROBiosystems (Beijing, China). Ninety-six-well microplates (Corning, USA) were coated with 1 μg/ml antigen proteins in carbonate bicarbonate buffer (pH 9.6), and the plates were incubated at 4 °C overnight. The plates were then blocked at 37 °C for 1 h with PBS (pH 7.4) in 2% bovine serum albumin (Sigma, USA) and washed with PBST. Serial dilutions of sera were added to the plates and incubated at RT for 1 h. HRP-conjugated goat anti-mouse IgG (Abcam, UK, 1:10,000 dilution) was added to the plates, and the plates were incubated at RT for 1 h and washed with PBST. The assay was developed for 10 min at RT with 100 μl of TMB substrate solution (Solarbio, China), stopped by the addition of 50 μl of stop solution (Solarbio, China) and then measured at 450 nm/630 nm (SPECTRA MAX 190, Molecular Device, USA). The endpoint titer was defined as the highest reciprocal serum dilution that yielded an absorbance ≥2.1-fold over the negative control serum values.

### Generation of pseudovirus and neutralization assay

SARS-CoV-2 pseudovirus bearing the full-length S protein of SARS-CoV-2 was produced in an Env-defective, luciferase-expressing HIV-1 backbone.^23^ Briefly, the S sequences of SARS-CoV-2 were synthesized and subcloned into the pCAGGS plasmid (Youbio, China). A total of 7 × 10^6^ HEK293T cells were seeded into a 10-cm plate and cotransfected with 23 μg of pNL4-3.Luc-R-E− and 1 μg of pCAGGS-S using the TurboFect transfection reagent (Thermo Scientific, USA). Forty-eight hours later, the supernatants were filtered and stored below −70 °C. In serum neutralization assay, samples were heat-inactivated at 56 °C for 30 min, pseudovirus was diluted and mixed with serial dilution of samples in 96-well plates. After 1 h incubation at 37 °C, ACE2-293T cells were seeded. Forty-eight hours later, the activity of luciferase was measured using a Luciferase Assay System (Promega). The EC_50_ neutralization titers were calculated as the reciprocal of the dilution for which luciferase activity reached half of that of the negative control using the Reed-Muench method. To calculate the GMT value, the seronegative samples were defined as half of the initial dilution.

### Live SARS-CoV-2 microneutralization assay

Serum samples were heat-inactivated at 56 °C for 30 min. After that, 100 TCID_50_ of SARS-CoV-2 strain was mixed well with serial dilution of samples in 96-well plates. After 1 h incubation at 37 °C, the mixture was transferred to Vero E6 cell monolayers. After 60 h incubation, the supernatant was removed and 0.05% crystal violet was added to stain the cells for 40 min, the decolorization solution was then added and OD values (570 nm/630 nm) were measured. The NAb titers were calculated using the Reed-Muench method to estimate the dilution of samples acquired for half-maximal OD values. To calculate the GMT value, the seronegative samples were defined as half of the initial dilution.

### Statistical analysis

The analysis was performed with GraphPad Prism v.7.00. Unpaired *t-*tests were conducted to compare differences between two experimental groups. One-way ANOVA with Tukey’s multiple comparisons tests were applied to compare more than two experimental groups. *p < 0.05, **p < 0.01, and ***p < 0.001 were considered to indicate significance. The antibody titer data were log transformed before analysis. The error bars throughout all the figures represent one standard deviation.

## Supplementary information

Supplementary Materials

## Data Availability

All data and methods in this study are available upon request.
