# Supplementary material for: Comparative characterization of antibody responses induced by Ad5-vectored spike proteins of emerging SARS-CoV-2 VOCs
Source: Signal Transduct Target Ther. 2022 Jul 29;7:257. doi: 10.1038/s41392-022-01065-0 (PMC9334975; doi:10.1038/s41392-022-01065-0)
Supplement: Supplementary file 1 — Supplementary Materials [file 41392_2022_1065_MOESM1_ESM.docx]

Supplementary Materials for

**Comparative characterization of antibody responses induced by Ad5-vectored spike proteins of emerging SARS-CoV-2 VOCs.**

Busen Wang^1,#^, Jinghan Xu^1,#^, Shipo Wu^1^, Zhe Zhang^1^, Zhenghao Zhao^1^, Jun Zhang^1^, Ling Fu^1^, Xiaodong Zai^1^, Yudong Wang^1^, Guanying Zhang^1^, ZhengShan Chen^1^, Yi Chen^1^, Hancong Sun^1^, Xiaohong Song^1^, Jinlong Zhang^1^, Lianhui Zhu^1^, Lihua Hou^1^,*, Wei Chen^1^,*

^1^ Beijing Institute of Biotechnology, No. 20 Dongdajie Street, Fengtai District, Beijing 100071, China.

^#^These authors contributed equally to this work.

*Correspondence to: [houlihua@sina.com](mailto:houlihua@sina.com) (L.H), [cw0226@foxmail.com](mailto:cw0226@foxmail.com,) (W.C)

**This PDF file includes:**  Figures. S1 to S7

**
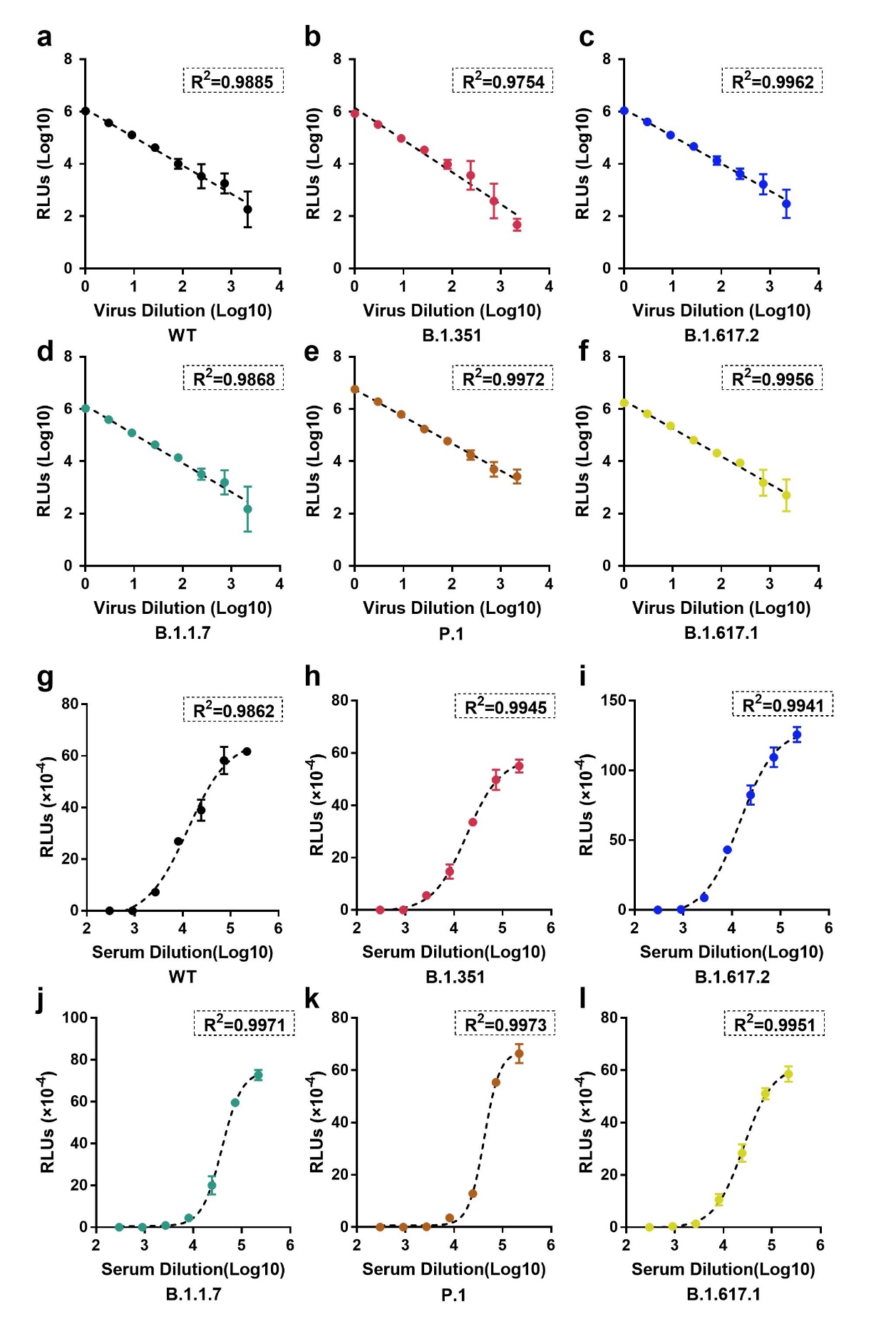
**

**Supplementary Figure S1 Validation of the pseudovirus neutralizing antibody detection method.** ACE2-293T cells were infected with serially diluted pseudoviruses of SARS-CoV-2-WT, B.1.351, B.1.617.2, B.1.1.7, P.1 and B.1.617.1. A linear correlation was found between virus dilution and luciferase activity (a-f) and R square was calculated using Pearson correlation analysis. A typical “S” type neutralizing curve was observed when pseudoviruses were neutralized by serially diluted positive serum (g-l), and were fitted using nonlinear regression with four parameters in Graphpad software.


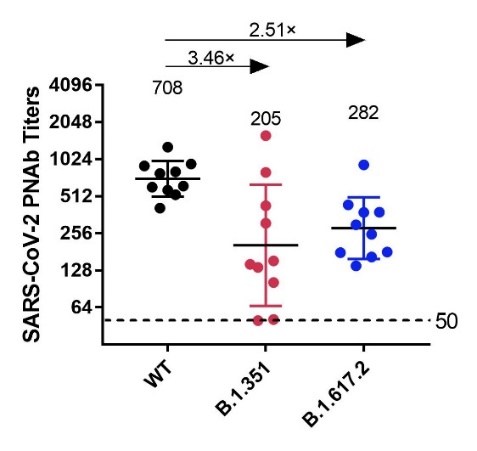


**Supplementary Figure S2 Pseudovirus neutralizing antibodies induced by a single dose of Ad5-WT.** The neutralizing antibodies against SARS-CoV-2-WT, B.1.351 and B.1.617.2 pseudoviruses in the Ad5-WT-immunized mouse group are shown.


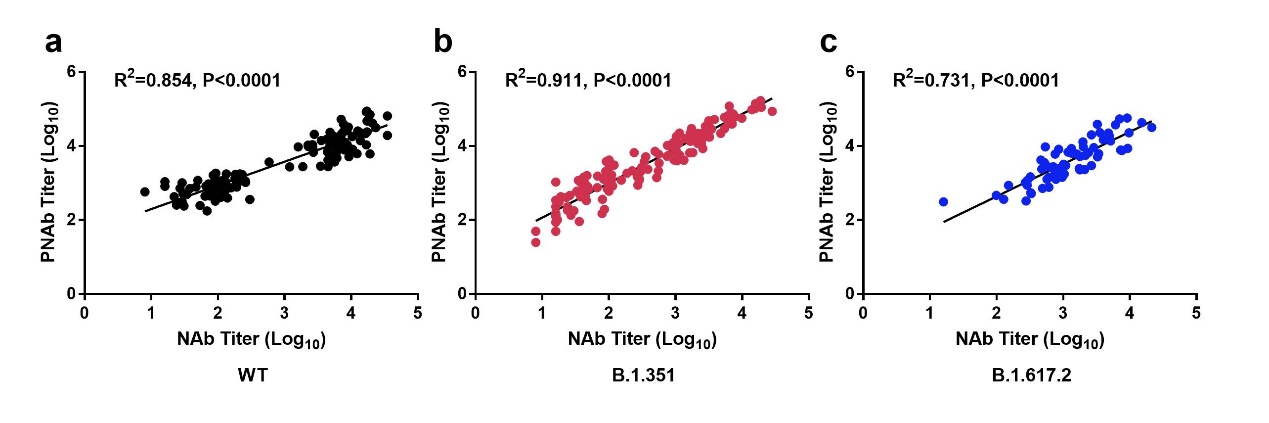


**Supplementary Figure S3 Correlation analyses between the PNAb titers and the live virus NAb titers obtained from single-dose or prime-boost vaccination for the WT, B.1.351 and B.1.617.2 strains.** The PNAb titers and the live virus NAb titers against the WT (a), B.1.351 (b) and B.1.617.2 (c) strain obtained from single-dose or prime-boost vaccination regimens with Ad5-WT, Ad5-B.1.1.7, Ad5-B.1.351, Ad5-P.1, Ad5-B.1.429, Ad5-B.1.617.1 and B.1.617.2 vaccines were taken together, and correlations between PNAb titers and NAb titers were calculated using Pearson correlation analysis.


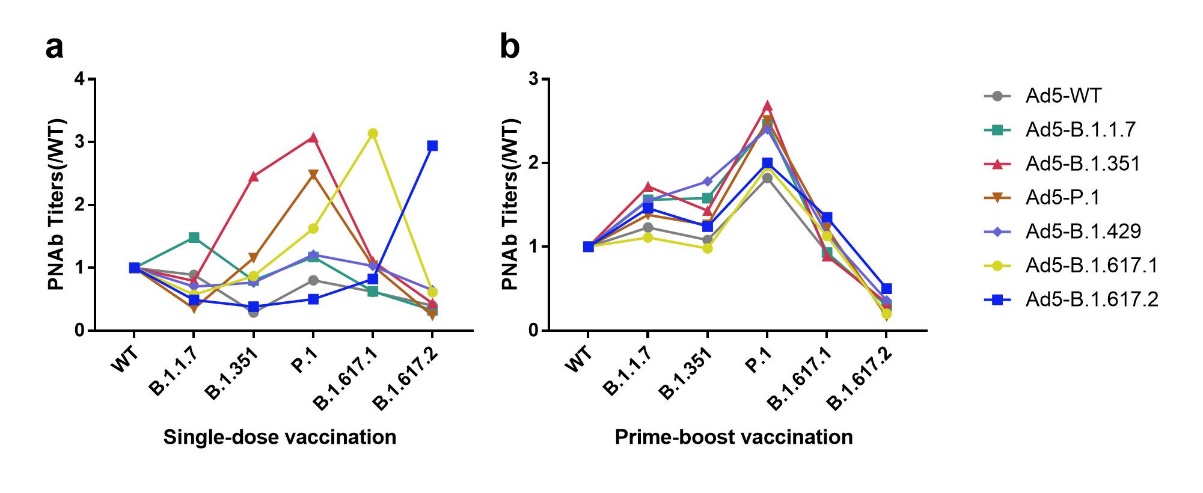


**Supplementary Figure S4 Neutralizing capacity of serum collected from the single-dose and prime-boost vaccination groups across diverse pseudoviruses.** The PNAb GMTs of serum at 28 days after the last immunization were normalized by dividing by those against the WT strain in the single-dose (a) and prime-boost (b) vaccination strategies.


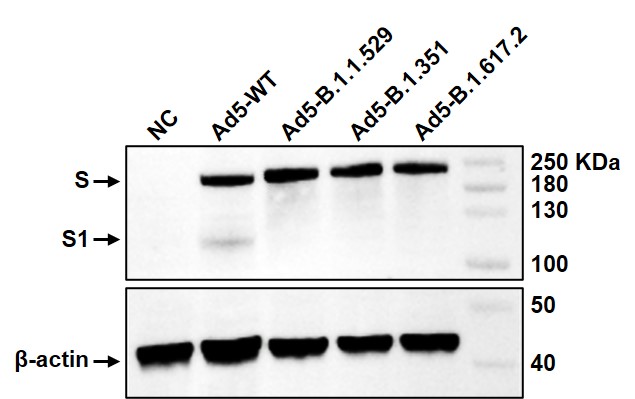


**Supplementary Figure S5 The transgene expression levels of the Ad5-WT, Ad5-B.1.1.529, Ad5-B.1.351 and Ad5-B.1.617.2 were validated through western blotting after infection in HEK293 cells.** HEK293 cells were infected with Ad5-WT, Ad5-B.1.1.529, Ad5-B.1.351, Ad5-B.1.617.2 or Ad5-null control (NC), and Western blotting was carried out at 24 h after infection.

**
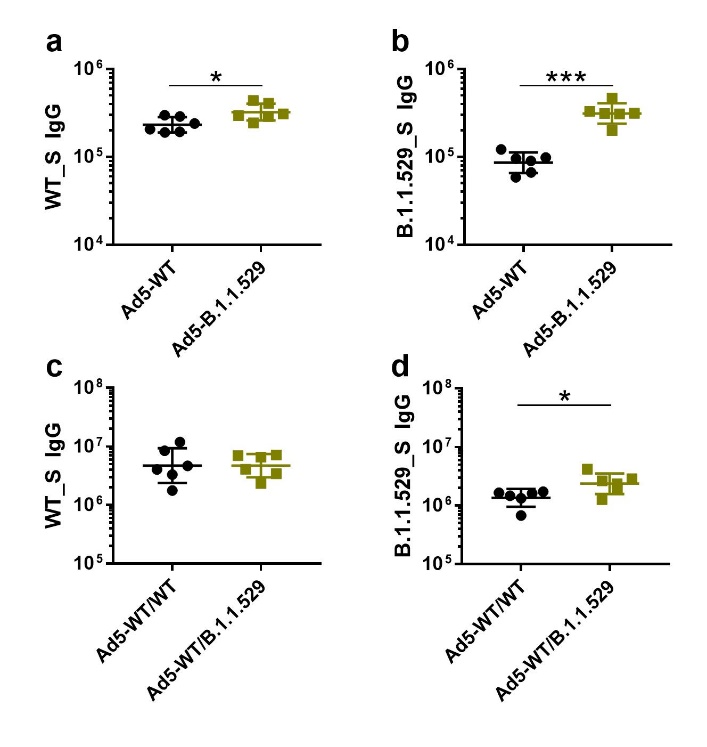
**

**Supplementary Figure S6 Serum IgG antibodies elicited by the Ad5-vectored spike protein of Omicron.** The serum IgG antibodies against WT or Omicron variant spikes at 28 days after immunization with a single dose of Ad5-B.1.1.529 or Ad5-WT are shown (a-b). Mice were primed with Ad5-WT and were boosted with Ad5-B.1.1.529 or Ad5-WT at 28 days after first vaccination. The serum IgG antibodies against WT or Omicron variant spikes at 28 days after last immunization are shown (c-d).

**
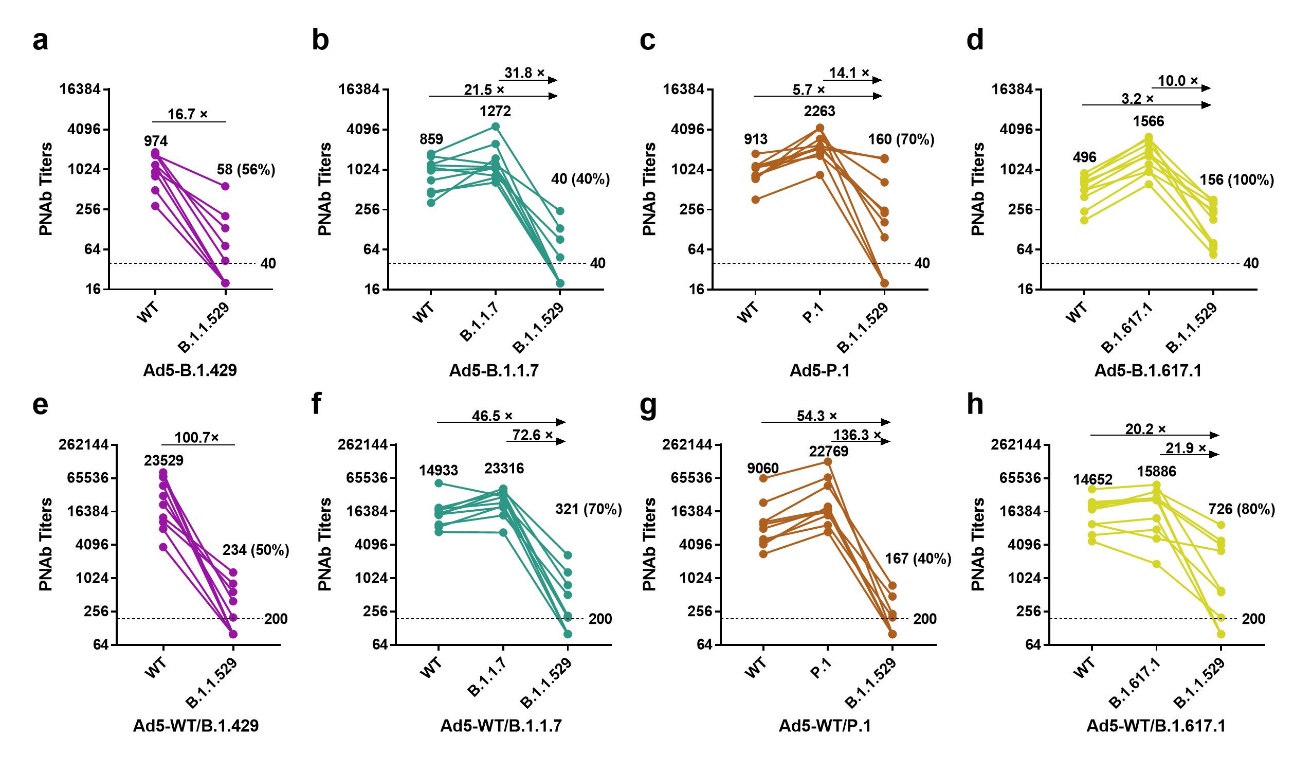
**

**Supplementary Figure S7 Cross-neutralization activities against the B.1.1.529 variant after the single-dose or prime-boost vaccination with the Ad5-B.1.429, Ad5-B.1.1.7, Ad5-P.1 and Ad5-B.1.617.1.** Mice were immunized with Ad5-variant vaccines based on a single-dose or prime-boost vaccination. The PNAb titers against the WT, self-matched and B.1.1.529 variant strains in the Ad5-B.1.429 (a and e), Ad5-B.1.1.7 (b and f), Ad5-P.1 (c and g), and Ad5-B.1.617.1 (d and h) vaccination groups were tested at 28 days after the last immunization.
